# Optimization of a solid-phase extraction step by experimental design for application to SPE-GC-ECD analysis of four bromobenzoquinones and 2,4,6-tribromophenol in chlorinated seawater

**DOI:** 10.1016/j.heliyon.2024.e40583

**Published:** 2024-11-20

**Authors:** Jean-Luc Boudenne, Carine Demelas, Laurent Vassalo, Bruno Coulomb, Julien Dron, Michelle Sergent, Etienne Quivet

**Affiliations:** a Aix Marseille Univ, LCE, Marseille, France; b Aix Marseille Univ, Avignon Université, CNRS, IRD, IMBE, Marseille, France; c Institut Ecocitoyen Pour la Connaissance des Pollutions, Centre de vie la Fossette RD 268, 13270, Fos-sur-Mer, France

**Keywords:** Brominated disinfection by-products, Halobenzoquinones, Chlorination by-products, Sample storage

## Abstract

Bromobenzoquinones and 2,4,6-tribromophenol belong to disinfection or chlorination by-products than can be formed in bromide-rich waters during chlorination or chloramination. Due to their high toxicities, sensitive and cost-effective analytical methods are necessary to detect and quantify them in various environmental matrices. A determination method of 2,5-dibromo-1,4-benzoquinone, 2,6-dibromo-3,5-dimethyl-1,4-benzoquinone, 2,6-dibromo-3-chloro-5-methyl-1,4-benzoquinone, 2,3,5,6-tetrabromo-1,4-benzoquinone and, 2,4,6-tribromophenol was developed using solid-phase extraction and electron capture detector-gas chromatography separation and detection (SPE-GC-ECD). Preservation of the four bromobenzoquinones with ascorbic acid allow to stabilize them into their bromohydroquinone analogues and to quench residual chlorine. Efficiency of different sorbents was tested and extraction and elution parameters were optimized by use of an experimental design. The recovery rates of each of the five compounds studied were between 59 and 101.4 %. The limits of detection (LODs) of the SPE-GC-ECD method were between 7 and 22 ng L^−1^. Applying this analytical procedure to real industrial chlorinated discharges in seawater, we report for the first time the presence of 2,6-dibromo-3- chloro-5-methyl-1,4-benzoquinone (up to 47 ng L^−1^), 2,6-dibromo-3,5-dimethyl-1,4-benzoquinone (35 ng L^−1^) and 2,4,6-tribromophenol (up to 42 ng L^−1^) in such effluents.

## Highlights

• Optimization by experimental design of the solid-phase extraction of halobenzoquinones in seawater.
• Storage and analysis of halobenzoquinones is highly improved by their conversion into their haloquinones analogues.
• Low limits of detection and quantification of halobenzoquinones and tribromophenol.
• A SPE-GC-ECD method allowing simultaneous determination of several families of DBPs.
• First reported occurrence of halobenzoquinones at the outlets of industrial plants.

## Graphical abstract

**Figure:**
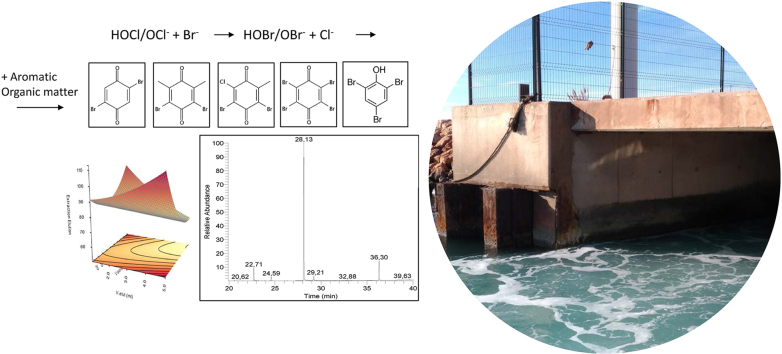

## 1 Introduction

Halobenzoquinones (HBQs) and bromophenols are considered emerging compounds posing environmental and/or health issues. The first group of compounds (HBQs) was identified relatively recently, first in drinking water [1,2], then in wastewater treatment plant discharges [3] and finally, in swimming pool waters [4]. These compounds are formed by the reaction between natural and/or anthropogenic organic matter, inorganic halides and chlorine (or chloramines) [5]. Phenols, chlorinated phenols, para-substituted phenolic compounds and parasubstituted aromatic amines serve as precursors of HBQs [6]. Recent studies have shown that HBQs are highly cytotoxic and/or induce greater developmental toxicity than most regulated disinfection by-products (DBPs) and are potentially genotoxic and carcinogenic [[7], [8], [9]]. 2,4,6-tribromophenol (TBP) has been present in the environment for longer, since it was first detected in the marine environment in the 1970s as a naturally-synthesized molecule by various marine organisms to defend themselves against predators and biofouling [10]. However, this molecule has recently attracted growing interest following its detection in several environmental compartments [11,12] linked to anthropogenic sources. TBP is indeed used as a pesticide and wood preservative and is known to be a degradation product of brominated flame retardants and polybrominated diphenyl ethers [13]. It has also been proven to be toxic to a number of marine organisms [11,14,15]. HBQs and TBP can also be formed in marine waters, particularly in areas where seawater chlorination is used to limit the formation of biofilm (micro-fouling) -that may block the heat exchangers- or to prevent the development of mold (macrofouling) in industrial pipes. This process is thus used at numerous industrial sites around the world to use this water for cooling (petrochemical and steel industries), heating (liquefied natural gas (LNG) terminals for the liquefaction of gases) or desalination [[16], [17], [18]]. This process has also been selected by the International Maritime Organisation as one of the methods to be used for the control and management of ships ballast water prior to discharge into the sea [19]. In all these cases, regulations generally require the identification of all chlorination by-products (CBPs) likely to be formed during the chlorination process [20]. The need for an analytical protocol using the same apparatus for the discrimination of several CBPs families is therefore important for laboratories and/or industrial managers. To date, several analytical methods have been developed for the quantification of HBQs and TBP in freshwaters, using Gas Chromatography-Mass Spectrometry (GC-MS) or Liquid Chromatography-Electrospray Ionization-Tandem Mass Spectrometry (LC-ESI-MS/MS), often coupled with a preconcentration step by liquid-liquid or solid-phase extraction, or using large-volume samples, enabling the quantification of HBQs and TBP at the ppt level [4,21]. Despite the low detection and quantification limits thus obtained, the use of mass spectrometry is not affordable for every budget and does not allow the analysis of all families of DBPs that may be found in aquatic environments. Electron capture detector-gas chromatography (GC-ECD) therefore seems more appropriate to meet these operational and economical needs, since this method has already proved its effectiveness in detecting DBPs in a single run, such as trihalomethanes, haloacetonitriles, halonitromethanes, haloanisoles and haloacetic acids (after derivatization) [22,23].In this paper, we propose a method for the quantification of four bromobenzoquinones and 2,4,6-tribromophenol in seawater, based on a solid-phase extraction (SPE) step followed by a GC-ECD analysis. This method was optimized through the use of an experimental design, including the study of the influence of several parameters (sample volume and flow rate through cartridge, and eluent volume and flow rate).In addition, in the context of environmental monitoring, including both sampling and analysis, a stability study was conducted over 100 h, with and without preservative agent (ascorbic acid) at two temperatures. The use of ascorbic acid not only quenches residual chlorine, but also stabilizes HBQs by reducing them to their halohydroquinones (HHQs) analogues. The whole procedure was applied to the detection of HBQs and TBP at the outlets of industrial chlorinated seawater.

## 2 Materials and methods

### 2.1 Reagents, chemicals, and materials

2,5-dibromo-1,4-benzoquinone (2,5-DBBQ), 2,5-dibromohydroquinone (2,5-DBHQ), 2,6-dibromo-3,5-dimethyl-1,4-benzoquinone (2,6-DBDMBQ), 2,6-dibromo-3-chloro-5-methyl-1,4-benzoquinone (2,6-DBCMBQ), 2,3,5,6-tetrabromo-1,4-benzoquinone (Tetra-BBQ), 2,4,5-trichlorophenol, 2,4,6-tribromophenol (TBP), Methyl tert-butyl ether (MTBE; for GC-ECD and GC-FID, Suprasolv®), and L-Ascorbic acid (99 %) were purchased from Sigma-Aldrich (Darmstadt, Germany). n-tetracosane-d50 was purchased from C/D/N Isotopes Inc. (Quebec, Canada). Stock solutions of HBQs and of 2,5-DBHQ were prepared by dissolving HBQs in MTBE in the range of 2–100 mg mL^−1^. Standard mixes were made monthly, stored at 10 °C, and monitored for changes in sensitivity.

Oasis HLB (200 mg, 6 mL) cartridges were bought from Waters Corporation (Milford, USA). Recovery™ C18 (500 mg, 6 mL) cartridges were purchased from Interchim (Montluçon, France).

Discovery SPE DSC-Si Silica Tube Supelco (1 g, 6 mL) cartridges were obtained from Sigma-Aldrich (Darmstadt, Germany). Cartridges were mounted in a VISIPREP SPE Manifold Supelco (Sigma-Aldrich (Darmstadt, Germany). Artificial seawater (ASW) was prepared according to ASTM D1141-98 [24].

### 2.2 Stability of HBQs in analytical seawater

To determine the stability of HBQs (Table 1) in the environment, sample aliquots (50 mL) of ASW containing the four HBQs at 2 mg L^−1^ were exposed to different conditions of temperature and acidification. To mimic environmental conditions, two temperatures, i.e. 9 °C and 20 °C, were selected.

**Table 1:**
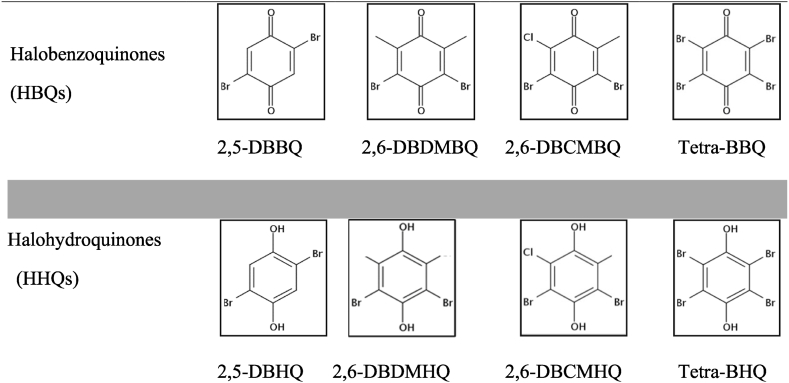
Structures of haloquinones under study: Halobenzoquinones (HBQs) and halohydroquinones (HHQs).

To improve the stability of environmental samples, experiments were conducted with or without the addition of 1 mL of ascorbic acid (AA) at 180 g L^−1^. The use of AA has the triple advantage of quenching residual chlorine (in case of sampling of chlorinated samples), decreasing the pH to 4 and transforming HBQs into HHQs (by redox reaction described in section 3) [25], which are more stable than their HBQ congeners (as also shown in section 3.2).

Each experiment was conducted in amber bottles. Samples were extracted at various times (from 0.5 h to 96 h), by liquid-liquid extraction (LLE) according to U.S. EPA Method 551.1 with slight modifications [22]. Sodium sulfate (10 g) was added, followed by MTBE (5 mL) and manuel shaking for 4 min. MTBE extraction rates for each HBQs and HHQs are given in the supplementary section (Table S1).

Then, 200 μL of the organic phase was collected for GC-MS analysis. The internal standard (n-tetracosane d50) was added to the samples (20 μL at 3 mg L^−1^ into 200 μL) and used as a surrogate. Each of the four conditions was performed in triplicate. The mean concentration and standard error were reported.

The time required to convert HBQs into HHQs in the presence of AA was determined using the simultaneous commercial availability of 2,5-DBBQ and 2,5-DBHQ. Complete conversion was achieved in 10 min, as determined by GC-MS analysis (see section 3.1). This duration was further considered sufficient for conversion of the three other HBQs into their HHQ analogues.

### 2.3 Characterization and quantification of haloquinones (HBQs + HHQs) by GC-ECD-MS

The GC-ECD-MS system consisted of a gas chromatograph coupled to a 63Ni electron-capture detector (GC-ECD model Clarus 580; PerkinElmer, Norwalk, CT, USA), and a Clarus SQ 8T (PerkinElmer, Norwalk, CT, USA). The system was composed of two injectors: one for the ECD and the other for the MS detector. This system made it possible to switch as often as necessary between the ECD or the EI-MS detector. The identification and the characterization of haloquinones were realized with MS, whereas their quantifications in environmental samples were realized with ECD.

The injection program, oven program, and column are the same for each detector. A PerkinElmer Elite 5MS capillary column (length 30 m, I.D 0.25 mm, film thickness 1 μm; PerkinElmer, Norwalk, CT, USA) was used for the separation. The injection volume was 1 μL and the injection temperature was set at 125 °C. Helium 5.0 was used as a carrier gas at 1 mL min^−1^. Nitrogen 5.0 was used as a make-up gas at 30 mL min^−1^. For the analysis of HBQs and HHQs, the temperature program was as follows: initially, 65 °C held for 5 min, then at a rate of 7 °C min^−1^ up to 220 °C, afterward 15 °C min^−1^ up to 240 °C and held for 8 min, and finally at 40 °C min^−1^ to 310 °C, held for 8 min.

The MS instrumental parameters were optimized as follows: electron impact 70 eV; source temperature 220 °C; transfer line 300 °C; data recorded in the full scan mode in the mass range (*m*/*z*) of 50–500 amu.

TurboMass software v6.1.0 was used for acquisition and data treatment.

For quality control, field blanks and laboratory blanks were sampled. Procedural standard calibration was performed using spiked seawater calibration standards, which were treated exactly in the same manner as the samples. Internal standard (2,4,5-trichlorophenol) was added to the samples at a final concentration of 100 μg L^−1^ and was used as a surrogate to monitor the reliability of the complete analytical procedures. A set of at least seven laboratory spiked standard solutions were analyzed to calculate the relative standard deviation (RSD). The detection limits (LODs) and quantification limits (LOQs) for the analysis of HBQs, HHQs and TBP were determined by GC-ECD. LODs and LOQs were estimated at 3 times and 10 times the signal-to-noise ratio, respectively. The relative standard deviation for each analyte was calculated by injecting seven replicates of a fortified solution at a concentration estimated to be near the LOD.

### 2.4 Experimental design and response surface methodology

Response surface methodology (RSM) consists of a group of mathematical and statistical techniques that can be used to establish the relationships between the outputs and independent inputs variables. In RSM, an empirical mathematical model is postulated, and a suitable experimental design is performed to estimate required coefficients. This model, once validated, can be used to predict the outputs in the whole experimental domain with a good precision and the visualization of the predicted response can be obtained by response surface plots (3D) or contour plots (2D).

To improve the extraction rates for each HHQs, this RSM was used to optimize each step and each parameter influencing the solid-phase extraction step (retention rates, extraction rates and global recovery rates). Firstly, some factors were fixed (Table 2). ASW was spiked to 500 μg L^−1^ to ensure the detection even if conditions were not favorable for the extraction. AA was added into ASW to reach pH 4 and to convert HBQs into their HHQ forms. As a first step, the experimental design was only applied to 2,6-DBDMHQ. Indeed, as the four HHQs have close chemical structures, the extraction parameters were also applied to the three other HHQs, i.e. 2,5-DBHQ, 2,6-DBCMHQ, and Tetra-BHQ and to TBP.

**Table 2:** Domains of variation of the parameters for the experimental design.

Domains of variation	
Fixed factors:	
Initial concentration:	500 μg L^−1^ (ASW)
Compound selected:	2,6-DBDMHQ
Sample pH:	4
Variable Factors	Experimental domain
Sample volume:	10 mL–200 mL
Sample flow rate:	0.5 mL min^−1^ - 3 mL min^−1^
Elution volume:	1 mL–5 mL
Elution flow rate:	0.5 mL min^−1^ - 3 mL min^−1^

Four parameters were tested: sample volume, sample flow rate, elution volume, and elution flow rate. The domains of variation for each factor were determined based on knowledge of the system (VISIPREP SPE Supelco Manifold) and the needs and constraints for the analysis in the environment.

A second-order polynomial model as presented in Eq. (1) was postulated to capture the possible non-linear effects and curvatures in the studied domain:

(1) *y* = *b*_0_ + *b*_1_*X*_1_ + *b*_2_*X*_2_ + *b*_3_*X*_3_ + *b*_4_*X*_4_ + *b*_11_ *×* _1_^2^ + *b*_22_ *×* _2_^2^ + *b*_33_ *×* _3_^2^ + *b*_44_ *×* _4_^2^ + *b*_12_ *×* *1 ×* _2_ + *b*_13_ *× 13 ×* _3_ + *b*_23_ *× 2 ×* _3_ + *b*_14_ *× 1 ×* _4_ + *b*_24_ *×* *2* *×* _34_ + *b*_34_ *×* *3* *×* _4_

Where Xj (j = 1, 2, 3, 4) were the unidimensional variables and b_0_, b_i_, b_ii_, and b_ij_ were regression coefficients for the intercept, linear, quadratic, and synergic, respectively.

To estimate the coefficients of this model, a 2D-optimal design of experiments, with 23 distinct experiments in the hypercube defined by the 4 input variables, was performed. Experiment #23 was replicated three times to estimate the variance of the experimental error (Table 3).

**Table 3:** Experimental design and experimental results for 2,6-DBDMHQ extraction on Recovery C18.

	Sample volume (mL)	Sample flow rate (mL min^−1^)	Elution volume (mL)	Elution flow rate (mL min^−1^)	Recovery rates (%)
	X_1_	X_2_	X_3_	X_4_	
Experiment 1	10	0.5	1	0.5	90
Experiment 2	200	0.5	1	0.5	55
Experiment 3	10	3	1	0.5	87
Experiment 4	200	3	1	0.5	107
Experiment 5	200	0.5	3	0.5	35
Experiment 6	105	3	3	0.5	77
Experiment 7	10	0.5	5	0.5	72
Experiment 8	200	1.75	5	0.5	58
Experiment 9	10	3	5	0.5	103
Experiment 10	200	1.75	1	1.75	104
Experiment 11	10	1.75	3	1.75	97
Experiment 12	105	0.5	5	1.75	44
Experiment 13	200	3	5	1.75	69
Experiment 14	10	0.5	1	3	57
Experiment 15	200	0.5	1	3	51
Experiment 16	10	3	1	3	62
Experiment 17	105	3	1	3	76
Experiment 18	200	3	3	3	80
Experiment 19	10	0.5	5	3	74
Experiment 20	200	0.5	5	3	27
Experiment 21	105	1.75	5	3	67
Experiment 22	10	3	5	3	107
Experiment 23	105	0.5	3	0.5	32
Experiment 24	105	0.5	3	0.5	27
Experiment 25	105	0.5	3	0.5	34

## 3 Results and discussion

### 3.1 GC-MS characterization

**Fig. 1:**
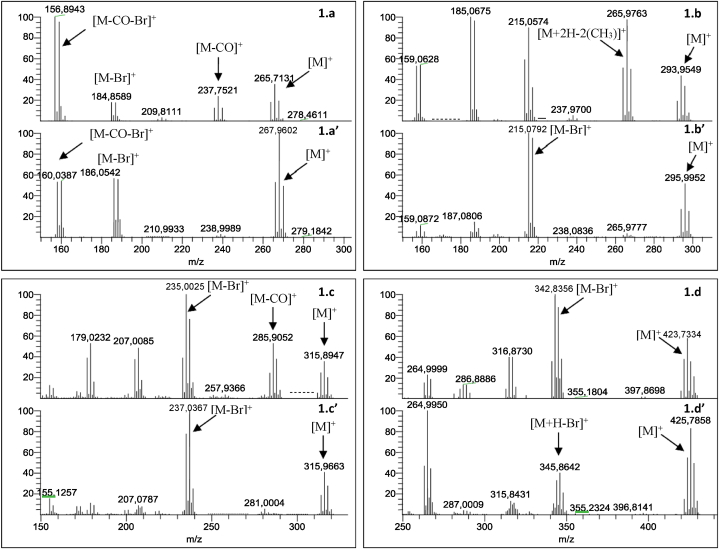
Mass spectrum measurements of the product ions of 2,5-DBBQ (1.a); 2,5-DBHQ (1.a’); 2,6- DBDMBQ (1.b) – 2,6-DBDMHQ (1.b’); 2,6-DBCMBQ (1.c) – 2,6-DBCMHQ (1.c’); Tetra-BBQ (1.d) –Tetra-BHQ (1.d’) in full scan mode in the mass range of 50–500 amu.

Firstly, the mass spectral characteristics of the four HBQs and HHQs were determined using an electron impact source, in full scan mode in the mass range from 50 to 500 amu. The mass spectra of each HBQ were determined by diluting stock solutions (prepared as described in section 2.1) to 10 mg L^−1^ for 2,6-DBDMBQ and 2,6-DBCMBQ, and to 100 mg L^−1^ for the other two. The mass spectrum of 2,5-DBHQ was determined in two ways: from the dilute solution of the commercially available product (see section 2.1), and after reduction of 2,5-DBBQ into 2,5-DBHQ by AA. Each compound was injected separately to identify its mass spectrum. Fig. 1 shows the differences obtained in the mass spectra of each HBQ compared with their HHQ congeners. Each compound was injected separately and directly in GC-MS. Regarding the HBQ forms, several similarities were able to be observed in their mass spectra. For the four HBQs, each molecular ion was detected [M]^+^.

**Fig. 2:**
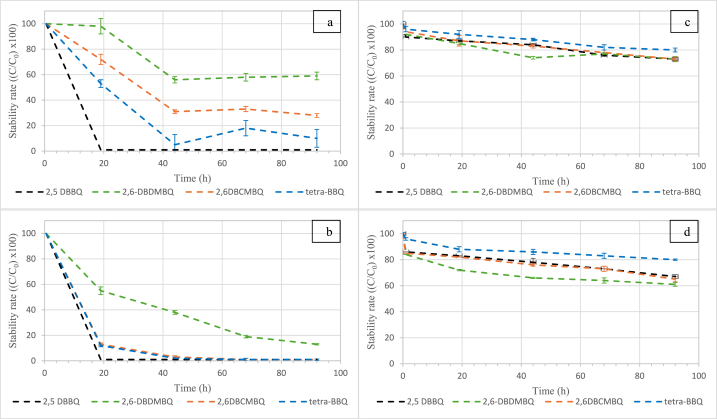
Stability in ASW (C_0_ = 2 mg L^−1^), at 9 °C (a,c) and 20 °C (b,d), without ascorbic acid (pH 8, 2a & 2b) and with ascorbic acid (pH 4, 2c & 2d), for 2,5-DBBQ, 2,6-DBDMBQ, 2,6DBCMBQ and tetra-BBQ.

The ionization of HBQs provoked the production of different fragments (four to five ions), such as [M − CO]^+^ for 2,5-DBBQ (Figs. 1a) and 2,6-DBCMBQ (Fig. 1c). Another fragment was commonly detected, corresponding to [M − Br]^+^: this fragment was present for 2,5-DBBQ (Fig. 1 a), 2,6-DBCMBQ (Fig. 1c) and Tetra-BBQ (Fig. 1d). In the case of 2,6-DBDMBQ (Fig. 1b), ionization provoked the loss of the two methyl groups [M + 2H–2(CH_3_)]^+^, corresponding to 2,6-DBBQ [C_6_H_2_Br_2_O_2_]^+^.

Regarding the HHQ forms (Fig. 1a’; Fig. 1b’; Fig. 1c’; Fig. 1d’), in each case, the molecular ion detected was [M]^+^. For each HHQs, the loss of one bromine was detected during ionization, with the production of a [M − Br]^+^ fragment. In contrast to HBQs, ionization of HHQs produced fewer fragments for each compound (two to three ions).

In situations where HBQ and HHQ forms were present together in the sample, the GC-ECD-MS method was developed to elute each haloquinone separately. Fig. S1 (in the Supporting Information) presents an example of the separation of an HBQ form (2,6-DBDMBQ) and an HHQ form (2,6-DBDMHQ).

### 3.2 Stability of halobenzoquinones in seawater

The stability of the four HBQs was separately studied in analytical seawater (ASW) for 4 days. Please note that stability of TBP was not studied in the present paper as this compound had already been proven to be stable in presence of AA [18]. For each HBQ at 2 mg L^−1^ as initial concentration, four conditions were tested: ASW temperature (20 °C or 9 °C) and with or without AA (1 mL at 180 g L^−1^; pH 4). Firstly, each HBQ was stored in the dark for 4 days, at 9 °C and 20 °C in ASW, without the addition of AA (pH 8). The results of these two conditions for the 4 HBQs are presented in Fig. 2 (on the left).

At 9 °C (Fig. 2a), after four days, 2,6-DBDMBQ, 2,6-DBCMBQ, and Tetra-BBQ still remained at (55 ± 3) %, (25 ± 2) %, and (10 ± 1) % of their initial concentration, respectively. On the other hand, 2,5-DBBQ was completely degraded within 19 h. At 20 °C (Fig. 2b), after four days, only 2,6-DBDMBQ remained at (10 ± 1) %. Both 2,6-DBCMBQ and Tetra-BBQ were fully degraded after 2 days, while 2,5-DBBQ, as at 9 °C, was fully degraded within 19 h. Under both conditions, 2,6-DBDMBQ was the most stable HBQ in ASW. Whatever the temperature conditions (9 °C or 20 °C), all four HBQs were not stable in ASW, with a degradation rate of at least 50 % in just 2 days. HBQs are indeed known to be hydrolysable in water to form halo-hydroxyl-benzoquinones, which are the more stable forms of HBQ DBPs in chlorinated waters [26]. To quantify HBQs in the environment, it is therefore necessary to stabilize them. AA is commonly used for the preservation of chlorinated waters as it both lowers pH and quenches residual chlorine [27]. However, the addition of AA (1 mL at 180 g L^−1^; pH 4) leads to the conversion of HBQs to their redox congeners (HHQs). Quinones are a class of molecules known for their ability to be reduced to their corresponding hydroquinones by AA [25], and the presence of electron-withdrawing groups, such as bromine groups, decreases the pKa and increases the E^0^ (HBQ/HHQ) [28]. For example, 2,5-DBBQ was transformed into 2,5-DBHQ, instantly after the addition of ascorbic acid.

Fig. 2 (on the right) presents the stability of HHQs after addition of ascorbic acid. At 9 °C (Fig. 2c) and at 20 °C (Fig. 2d), all HHQs were still present after 4 days, with a degradation rate lower than (26 ± 2) % and (38 ± 2) %, respectively. In these acidified conditions (pH 4), for both temperatures, Tetra-BHQ was the most stable HHQ in ASW (degradation rate: (20 ± 1) % at 9 °C and (21 ± 1) % at 20 °C) as compared to the three other HHQs (Fig. 2d). Thus, on the one hand, the addition of AA was needed to prevent disappearance of HBQs, but, in another hand, this addition converted them into HHQS. Therefore, with the addition of AA, HBQs were not able to be analyzed directly. It was thus necessary to develop an analytical method of quantification for global HHQs, i.e. the HHQs already present in the medium and the HHQs resulting from the transformation of HBQs by acidification of ASW. As these compounds are supposed to be present in the environment in low concentration (ng L^−1^) in seawater, a new method of solid-phase extraction (SPE) was developed, and each parameter was optimized with an experimental design.

### 3.3 Optimization and experimental design methodology for solid-phase extraction of haloquinones and 2,4,6-tribromophenol

**Fig. 3:**
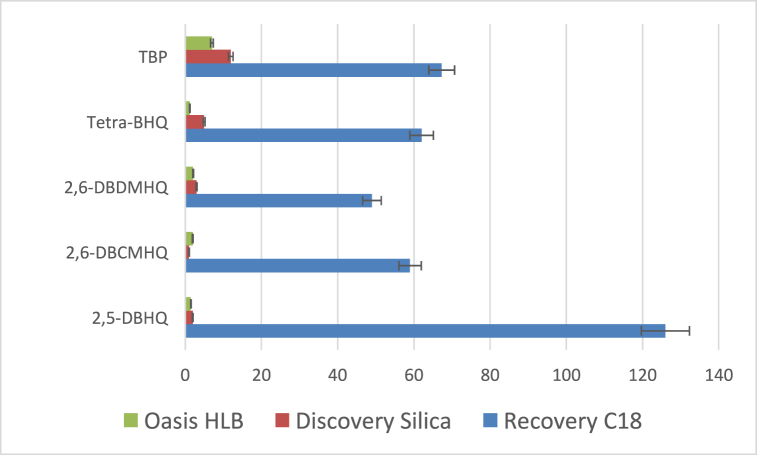
Extraction rates for the 4 HHQs and TBP as a function of cartridge used (Recovery C18, Discovery Silica, Oasis HLB).

To quantify HHQs and TBP in seawater, a preliminary experiment was performed to select the best solid phase for their extractions between three different cartridges. The three different cartridges were among the most used in the literature, i.e Recovery C18, Discovery Silica, and Oasis HLB [3,21,29,30]. These cartridges were mounted in a VISIPREP SPE Manifold (Supelco) and tested under the following conditions: sample volume: 200 mL of ASW (pH 4); HHQ concentration: 1 mg L^−1^; internal standard (2,3,5-trichlorophenol): 1 mg L^−1^. The sample and elution flow rates were controlled using a peristaltic pump.Each column was conditioned as follows: activation with CH_3_OH (6 mL at 0.5 ml min^−1^) and conditioning with ultrapure water at pH 4.0 at 0.5 ml min^−1^. After flowing 200 mL sample, the columns were rinsed with 6 mL ultrapure water (acidified at pH 4.0) at a flow rate of 3 mL min^−1^ and then eluted with MTBE (6 mL at 3 mL min^−1^). The extracts were then dried with 1.3g Na_2_SO_4_, before analysis by GC-ECD.Fig. 3 shows that percent recoveries for the 4 HHQs and TBP carried out with the same experimental conditions varied considerably from one cartridge to another. Indeed, whatever the HHQs, Discovery Silica, and Oasis HLB cartridges had the lowest extraction rates (lower than 12 % for each compound). Recovery C18 cartridges showed the best results with recovery percentages ranging from 49 ± 7 % for 2,6-DBDMHQ to 126 ± 17 % for 2,5-DBHQ. Consequently, recovery optimization was conducted with this SPE cartridge (500 mg, 6 mL) using an experimental design. The experimental conditions provided by the experimental design are described in Table 3 along with the various experimental results.

For the different experimental conditions, recovery percentages ranged from 27 to 107 %, suggesting that the parameters studied were influential in this domain of variation. Based on these results, the coefficients of the postulated model were estimated using multilinear regression and are presented in Eq. (2). The calculations were performed with AZURAD software, developed for the construction and the treatment of experimental designs.

(2) *y* = 75.33 − 8.32 *X*_1_ + 18.31 *X*_2_ − 3.52 *X*_3_ − 1.18 *X*_4_ + 13.11 × _1_^2^ − 16.23 × _2_^2^ + 3.78 × _3_^2^ − 6.65 × _4_^2^ + 5.39 × 1 × _2_ − 14.18 × 1 × _3_ + 1.05 × 2 × _3_ + 1.06 × 1 × _4_ − 2.47 × 2 × _34_ + 3.33 × 3 × _4_

To validate the model suitability, several techniques were used: residual analysis, ANOVA (ANalysis Of VAriance), and prediction error sum of squares residuals (coefficient of determination, r^2^).All these tools led us to conclude that the regression models were significant: the changes in the selected responses could be explained by variation in the factors. Thus, the regression models could be considered reliable for predicting recovery percentages over the range of operating conditions tested.

To visualize the behavior of the response, response surfaces were graphically represented. Percent recoveries were plotted in terms of sample volumes (X_1_) and sample flow rates (X_2_) versus elution volumes (X_3_) and elution flow rates (X_4_). These contour plots (Fig. S2) show the elution flow rate had no preponderant effect on this response in the range studied. In contrast, we could observe an important influence of the three other parameters and the experimental design made it possible to highlight interaction effects between these factors: the influence of a parameter could change depending on the conditions of the other parameters. For instance, we could observe the percent recovery increases for a high sample volume when the elution volume was low and, when the elution volume was high, the sample volume had to be low. In addition, in these two maximization zones, it could be observed that the optimal value for the sample flow rate was about 2–2.5 mL min^−1^.

**Fig. 4:**
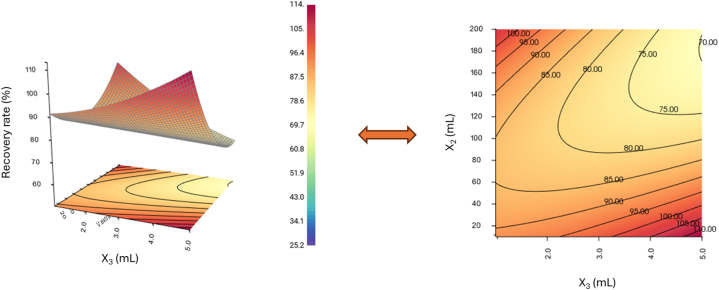
Response surface in the plane obtained for the Solid-Phase Extraction of 2,6-DBDMHQ: elution volume (X_3_), sample volume (X_4_), showing the 2 zones of optimal conditions (sample flow rate = 2 mL min^−1^, elution flow rate = 3 mL min^−1^).

In summary, the percentage recoveries were maximized in two domains (Fig. 4): the response increases for a high sample volume and a low elution volume, or for a low sample volume and a high elution volume.

In our case, there was a need to have the highest preconcentration factor allowing the quantification of HHQs in the environment. The first, corresponding to a low extraction volume (10 mL of seawater) for a high elution volume (5 mL of solvent) (bottom corner right in Fig. 4), allowed only a preconcentration factor equal to 2. So, it was necessary to choose the second domain where the recovery rates were maximized: a high sample volume (200 mL of seawater) and a low elution volume (1 mL of solvent) (top corner left in Fig. 4). With these conditions, we were able to obtain a preconcentration factor equal to 200. So, for the rest of the study, we used these parameters for the SPE method: 200 mL of the sample with an elution flow rate of 2 ml min^−1^ and 1 mL of solvent at an elution flow rate of 1.75 mL min^−1^.

### 3.4 Validation of the SPE-GC-ECD method

The method was optimized for the extraction of the 4 HHQs and TBP from seawater and to quantify them, the whole SPE method was coupled with GC-ECD (depiction of the whole procedure presented in the Supporting Information – Fig. S3). This combination of the SPE with the GC-ECD method enabled the quantification of the four HHQs at ng L^−1^ levels in seawater. The SPE-GC-ECD method was validated by the analysis of ASW spiked with the standards at 100 ng L^−1^ (n = 10). The recovery, limits of detection and limits of quantification of the whole method -determined in ASW- were presented in Table 4. Our method permits good reliability for each compound, with RSD between 4 % and 14 %, and recovery within a range from 59 % to 100 % for the four HHQs (Table 4). The LOQ obtained with this method for each compound was higher than others conducted by LC-MS/MS [21,30], but our GC-ECD method is the first one to be applied on seawater samples. Moreover, the interest of this GC-ECD method relies on the analysis of five other families of CBPs with the same apparatus [17,18].

**Table 4:** SPE recovery, limits of detection (LOD), quantification (LOQ) and precision of the SPE-GC-ECD method.

Chemical (μg L^−1^)	LOD	LOQ	RSD (%)	Recovery (%)	Range	Linearity (r^2^)
2,5-DBHQ	0.022	0.074	8	96	0.05–25.0	0.998
2,6-DBDMHQ	0.014	0.023	4	100.2	0.02–20.0	0.995
2,6-DBCMHQ	0.017	0.021	5	59	0.03–50.0	0.993
Tetra-BHQ	0.014	0.047	14	83	0.03–50.0	0.997
TBP	0.007	0.012	3	101.4	0.04–40.0	0.997

Potential matrix interferences were also studied by plotting a calibration curve for each compound in real seawater samples, taken from Gulf of Fos (Mediterranean Sea), far away from any industrial discharges. These samples were spiked with target molecules concentrations ranging from 50 to 2000 ng L^−1^. The analytical features obtained with these real samples were the same as those obtained with ASW, showing that there was no interference with seawater (Fig. S4).

### 3.5 Analysis of haloquinones and TBP at the industrial outlets of chlorinated seawater

Having determined optimum conditions for the sample preservation, the SPE step, and the detection by GC-ECD for each compound, chlorinated seawater samples were collected during summer 2019. The study site was in the Gulf of Fos, located in Southeastern France on the Mediterranean at about 50 km from the city of Marseille. The Gulf of Fos receives several freshwater inputs including a main input from the Rhône River and minor inputs from the Berre Lagoon and navigation canals (see map -Fig. S5- in supporting information). Various heavy industrial activities [17] are established around the Gulf including two large LNG terminals (designated by sampling stations 8X and 12X on the map) and one power plant (9X). During that sampling campaign, LNG terminals and power plant discharged chlorinated waters at a flow of 5400 m^3^ h^−1^, 28,500 m^3^ h^−1^, and 0 m^3^ h^−1^, respectively, generated by electrochlorination or addition of sodium hypochlorite (1.5 mg L^−1^ Cl_2_). Another sampling point (6P) was added to compare levels of the targeted molecules between industrial discharges and area not influenced by these kinds of discharges.

For the analysis of HBQs and TBP, samples were placed in amber glass bottles (1 L) where 10 mL AA (180 g L^−1^) were rapidly added. Bottles were sealed with PTFE-lined screw caps. These samples were filled without headspace to avoid any loss due to volatilization. Samples were stored at 4 °C in the dark and extracted by SPE for HBQs and TBP determination, not less than 24 h after the end of sampling.

During this sampling campaign, only two HHQs were detected in measurable concentration: 2,6-DBDMHQ was measured at 35 ng L^−1^ only at sampling site 8X, whereas 2,6-DBCMHQ was detected at sampling sites 8X and 12X at 47 and 25 ng L^−1^, respectively (Table 5). TBP was also detected in these two sampling sites at 15 and 42 ng L^−1^, respectively.

**Table 5:** Levels of halobenzoquinones and tribromophenol at the outlets of industrial chlorinated discharges.

Compound (ng L^−1^)/sampling sites	8X2	12X	9X	6P
2,5-DBHQ	–	–	–	–
2,6-DBDMHQ	35	–	–	–
2,6-DBCMHQ	47	25	–	–
Tetra-BHQ	–	–	–	–
TBP	15	42	–	–

These compounds were not detected in the sampling site 6P, indicating that their presences were linked to the industrial chlorinated discharges. Their formation seemed thus to be linked to the reaction between bromide (naturally present in seawater), added chlorine and organic matter (naturally and/or anthropogenically present). These kinds of mechanisms were already pointed out in the field of drinking waters, as these compounds are also known as disinfection by-products [[1], [2], [3],^6^,^7^,^21^].

## 4 Conclusions

This study showed the importance of sample preparation and stabilization for a good analysis of HBQs and HHQs in seawater. Indeed, the four HBQs were not stable over time, with degradation rates between 50 % and 100 % at 9 °C after two days, for each HBQs. The same consideration applied to the four HBQs at 20 °C, with degradation rates between 60 % and 100 % after two days. It was thus necessary to transform them into their HHQs form, by addition of ascorbic acid in the sample of seawater. Halohydroquinone form was more stable over times, with degradation rates between only 20 and 40 % after four days. This work highlights the optimized parameters for the quantification of these compounds in the environment. Indeed, this SPE-GC-ECD method is able to quantify the four HHQs and TBP at ng levels, with good reliability. Moreover, the use of AA also allows to quench residual chlorine and to stabilize the other CBPs.

Beyond the analytical aspects issued from this study, nature and level of CBPs, found in chlorinated seawater outlets into the sea, poses the question about the impacts of such discharges on marine fauna and flora.

## CRediT authorship contribution statement

**Jean-Luc Boudenne:** Writing – review & editing, Writing – original draft, Validation, Supervision, Resources, Project administration, Investigation, Formal analysis, Conceptualization. **Carine Demelas:** Data curation, Formal analysis, Investigation, Visualization. **Laurent Vassalo:** Investigation, Formal analysis, Writing – original draft. **Bruno Coulomb:** Data curation, Writing – review & editing. **Julien Dron:** Project administration. **Michelle Sergent:** Methodology. **Etienne Quivet:** Supervision, Conceptualization, Validation, Writing – original draft.

## Date availability statement

Data included in article/supplementary material/referenced in article.

## Declaration of competing interest

The authors declare that they have no known competing financial interests or personal relationships that could have appeared to influence the work reported in this paper.
