# Supplementary material for: Optimization of a solid-phase extraction step by experimental design for application to SPE-GC-ECD analysis of four bromobenzoquinones and 2,4,6-tribromophenol in chlorinated seawater
Source: Heliyon. 2024 Nov 20;10(23):e40583. doi: 10.1016/j.heliyon.2024.e40583 (PMC11625260; doi:10.1016/j.heliyon.2024.e40583)
Supplement: Multimedia component 1 [file mmc1.docx]

**Supporting Information for**

“Optimization of a Solid-Phase Extraction step by experimental design for application to SPE-GC-ECD analysis of four bromobenzoquinones and 2,4,6-tribromophenol in chlorinated seawater”

in *HELIYON Environment*

# Jean-Luc Boudenne^a,*^, Carine Demelas^a^, Laurent Vassalo^a^, Bruno Coulomb^a^, Julien Dron^c^, Michelle Sergent^b^, Etienne Quivet^a^,

# ^a^Aix Marseille Univ, LCE, Marseille, France

# ^b^Aix Marseille Univ, Avignon Université, CNRS, IRD, IMBE, Marseille, France

# ^c^Institut Ecocitoyen Pour la Connaissance des Pollutions, Centre de vie la Fossette RD 268, 13270 Fos-sur-Mer, France

# * Corresponding author. Phone +33-(0)413-551-031; Fax +33-(0)413-551-060; e-mail: jean-luc.boudenne@univ-amu.fr

Table S1. LLE-GC-MS method performance characteristics

Figure S1. GC-MS chromatograms of 2,6-DBDMBQ and 2,6-DBDMHQ

Figure S2. Contour plots of elution flow rate and elution volume

Figure S3. Depiction of the global procedure for the analysis of halobenzoquinones and 2,4,6-tribromophenol in seawater samples

Figure S4. Calibration curves of the halobenzoquinones and 2,4,6-tribromophenol obtained in real seawater samples

Figure S5. Map and photos of sampling sites

**Table S1.** LLE-GC-MS method performance characteristics

| Analyte | | Recovery rate (%) (n=3) | Precision (%) (n=3) | Linearity (r^2^) | Range (µg L^-1^) |
| --- | --- | --- | --- | --- | --- |
| HBQs | 2,5-DBBQ | 92 | 6 | 0.997 | 500 - 2000 |
|  | 2,6-DBDMBQ | 57 | 14 | 0.994 | 200 -2000 |
|  | 2,6-DBCMBQ | 91 | 10 | 0.992 | 300 -2000 |
|  | Tetra-BBQ | 97 | 8 | 0.996 | 500 - 2000 |
| HHQs | 2,5-DBHQ | 96 | 6 | 0.990 | 50 -2000 |
|  | 2,6-DBDMHQ | 64 | 12 | 0.996 | 50 - 2000 |
|  | 2,6-DBCMHQ | 95 | 8 | 0.992 | 40 - 2000 |
|  | Tetra-BHQ | 98 | 5 | 0.994 | 50 - 2000 |

**Figure S1.** Chromatograms of 2,6-DBDMBQ (RT: 22.71 min) and 2,6-DBDMHQ (RT: 28.13 min) obtained after LLE and injected on GC- MS. Internal standard (n-Tetracosane d-50) was eluted at 36.30 min.


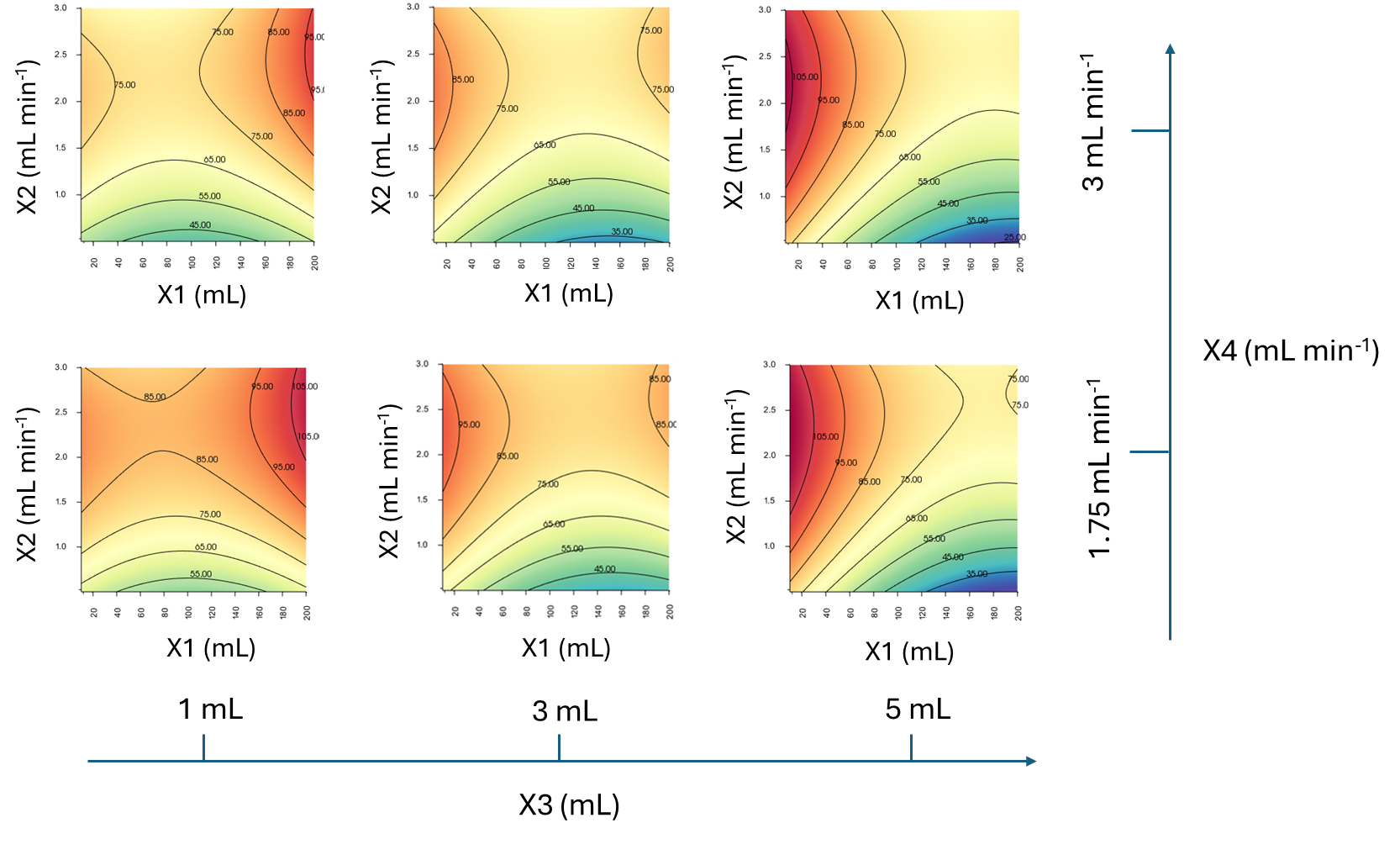
**Figure S2.** Contour plots of elution flow rate and elution volume

Percent recoveries were plotted in terms of sample volumes (X1) and sample flow rates (X2) *versus* elution volumes (X3) and elution flow rates (X4)

**
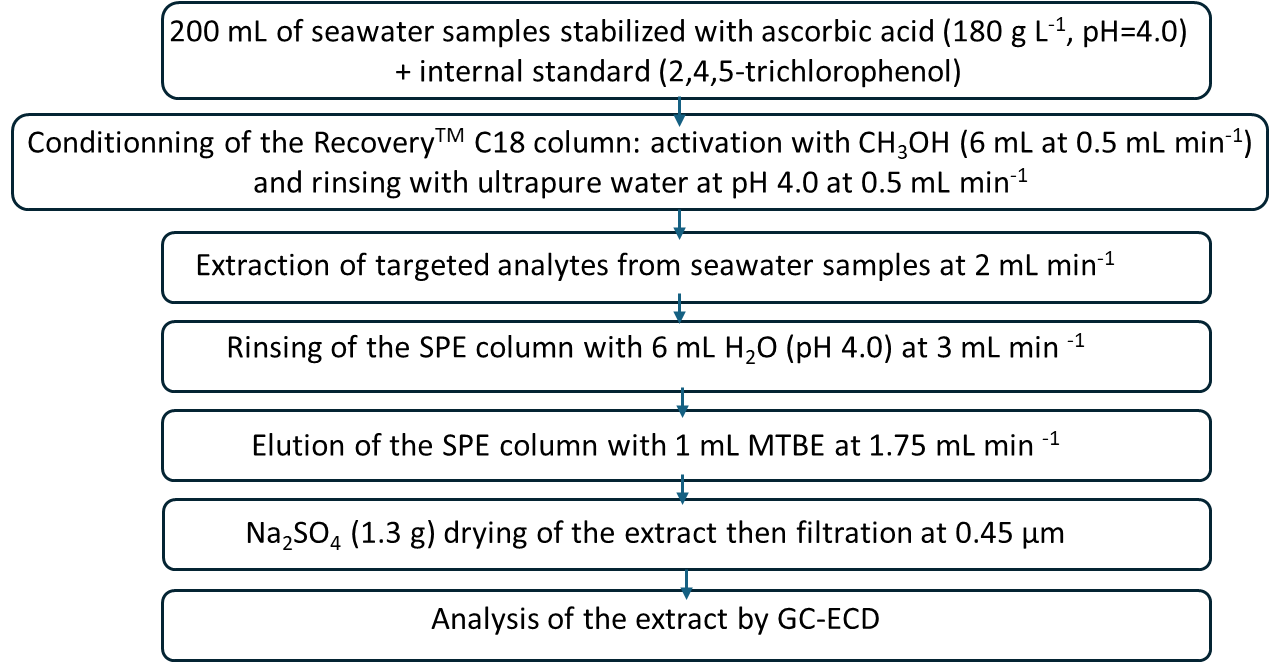
**

**Figure S3.** Depiction of the global procedure for the analysis of halobenzoquinones and 2,4,6-tribromophenol in seawater samples

**
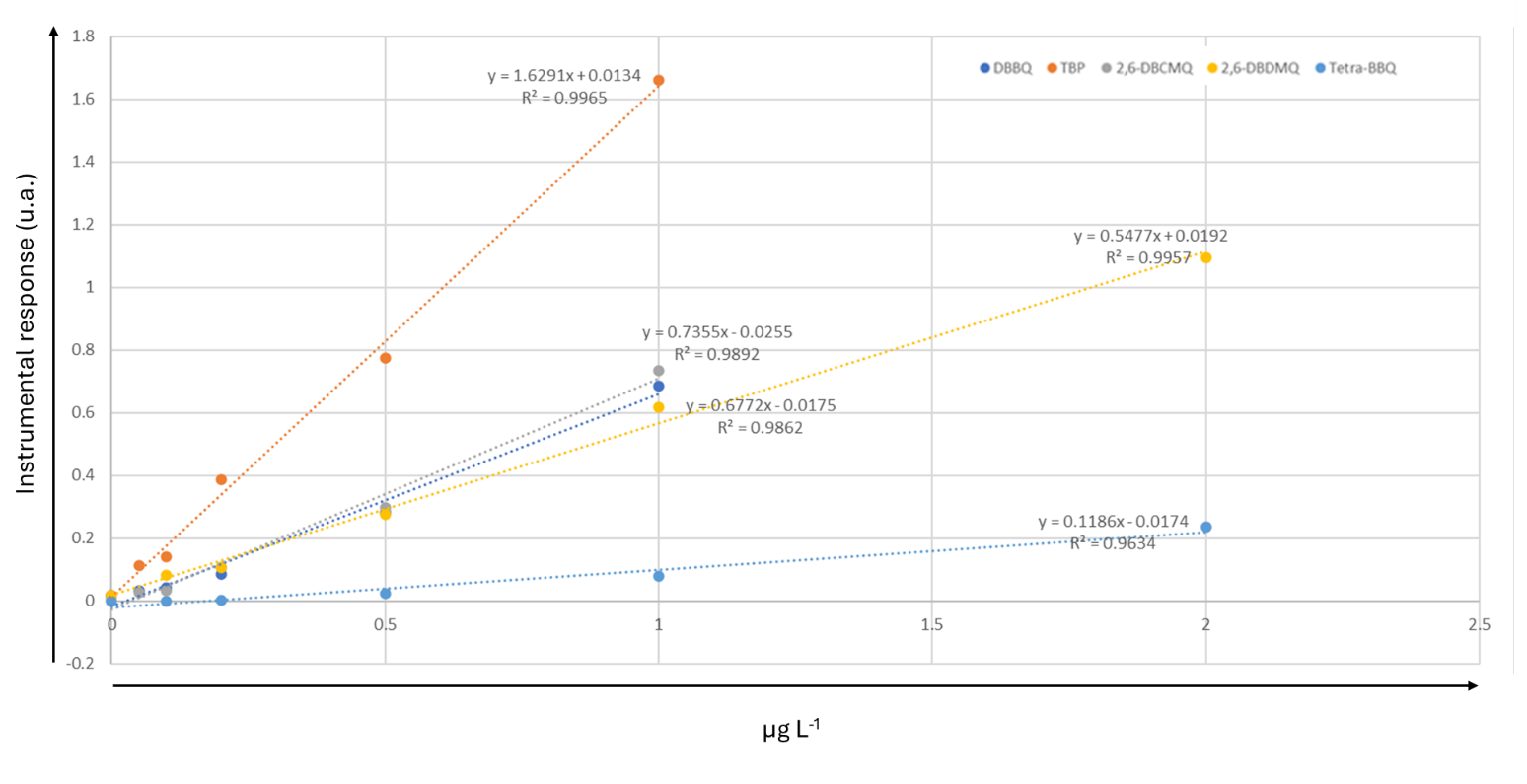
**

**Figure S4.** Calibration curves of the halobenzoquinones and 2,4,6-tribromophenol obtained in real seawater samples

**
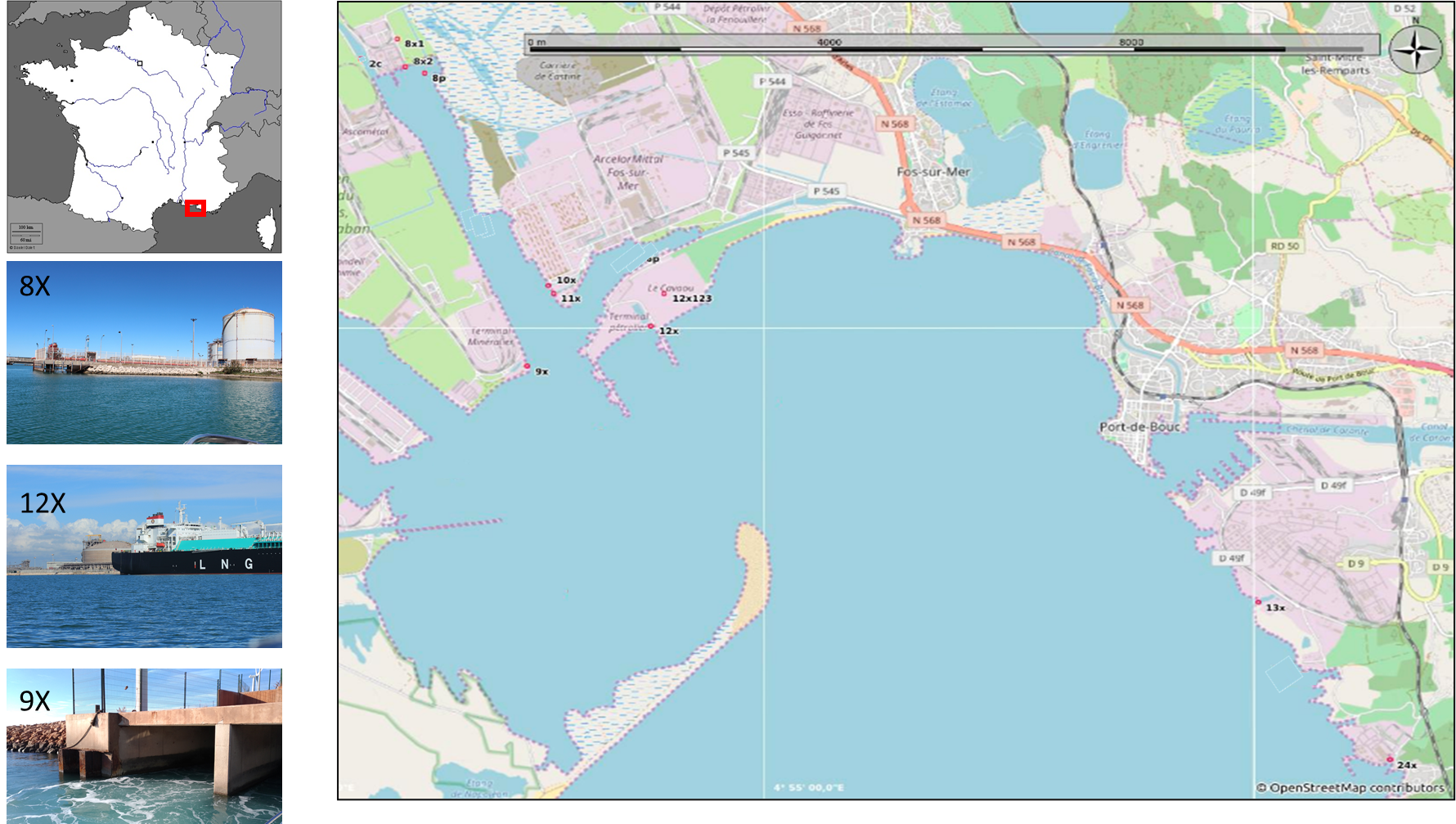
**

**Figure S5.** Map and photos of sampling sites (Gulf of Fos, Mediterranean Sea)
